# Effects of Different Pretreatments With Vacuum Drying on Drying Kinetics, Bioactive Properties and Physicochemical Characterization of Red Beetroot Snacks

**DOI:** 10.1002/fsn3.72234

**Published:** 2026-08-13

**Authors:** Nuray İnan‐Çınkır

**Affiliations:** ^1^ Department of Food Technology, Faculty of Kadirli Applied Science Osmaniye Korkut Ata University Osmaniye Türkiye

**Keywords:** betalains, infrared, microwave, pretreatments, red beetroots, ultrasound, vacuum drying

## Abstract

Innovative approaches for developing nutritious and healthier snack options from vegetable sources have become increasingly important due to modern dietary patterns and growing health concerns. This study aimed to evaluate the effects of various pretreatments (freezing [FP], infrared [IP], microwave [MWP], and ultrasound [USP]) on the vacuum drying kinetics, bioactive and physicochemical properties of red beetroot snacks. MWP significantly shortened the drying time to 290 min. The Sigmoid and Cubic equations were the best describing drying models. The highest *D*_eff_ was 2.52 × 10^−8^ m^2^/s in MWP. While USP provided the highest betalain content (8503.22 mg/kg), FP had the lowest value (3162.43 mg/kg). Betanin, isobetanin and vulgaxanthin I as betalain compounds were identified. MWP had the highest total phenolic content (6402.93 mg/kg), and the highest antioxidant activity (59.38%) was found in NP (no pretreatment). Dried red beetroots had softer texture for both pretreatments. All samples showed porous structures except for NP and FP. FP affected thermal behavior and functional groups of samples. Results showed that USP and MWP for vacuum drying provided better quality and physicochemical characteristics.

## Graphical abstract

Different pretreatments significantly affected the drying kinetics and quality attributes of vacuum‐dried red beetroot snacks. Microwave pretreatment markedly shortened the drying time and enhanced effective moisture diffusivity, while ultrasound pretreatment preserved the highest betalain content. Comprehensive physicochemical, structural, textural, thermal, and functional analyses demonstrated the beneficial effects of these pretreatments on product quality. The findings highlight the potential of microwave and ultrasound pretreatments as effective strategies for producing high‐quality, nutrient‐rich red beetroot snacks.

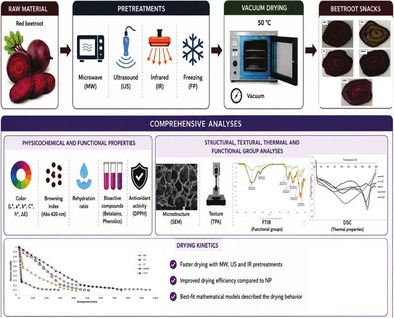

## 1 Introduction

Red beetroot ( *Beta vulgaris* L.) has recently attracted considerable attention as a functional food due to its rich content of bioactive compounds and associated health‐promoting properties. It contains significant levels of betalains, polyphenols, flavonoids, saponins, and carotenoids (Nemzer et al. 2011; Zhang et al. 2021; Liu et al. 2024). These bioactive compounds have been reported to exhibit various physiological benefits, including antioxidant, anti‐inflammatory, antihypertensive, lipid‐lowering, antidiabetic, anticancer, and antiviral activities (Nistor et al. 2017; Fu et al. 2020; Hadipour et al. 2020; Liu et al. 2024).

Betalains, the main pigment in beetroot, are divided into betacyanins (red‐violet pigments) and betaxanthins (yellow pigments) (Tarasevičienė et al. 2024). Their concentration is influenced by several factors, including cultivar, growing conditions, geographic origin, and postharvest storage conditions (Nistor et al. 2017; Fu et al. 2020; Liu et al. 2024). However, fresh beetroot is highly perishable due to its high moisture content, enzymatic activity, and microbial load, which significantly limits its shelf life. Drying is an effective preservation technique that reduces moisture content, thereby extending shelf life, decreasing product volume and weight, and improving storage and transport stability (Liu et al. 2024). Nevertheless, conventional drying processes may cause significant losses of heat‐sensitive bioactive compounds such as betalains. Therefore, maintaining the stability of these compounds during processing is a key challenge. Previous studies have shown that dried matrices can provide better protection for betalains against environmental factors such as pH, light, and temperature compared to fresh tissues, mainly due to reduced water activity and enzyme inactivation (Nistor et al. 2017; Tarasevičienė et al. 2024).

Drying method plays a crucial role in determining the final quality of beetroot products. The studies have investigated the effects of different drying techniques on physicochemical and functional properties of beetroot (Nistor et al. 2017; Liu et al. 2022; Tarasevičienė et al. 2024). Among them, vacuum drying (VD) has gained attention due to its low oxygen environment and relatively lower processing temperature compared to conventional drying methods, allowing better retention of nutritional and structural properties (Liu et al. 2021; Mella et al. 2022). However, its relatively long drying time remains a limitation. To overcome this limitation, various pretreatment techniques such as microwave, ultrasound, infrared, and freezing have been applied prior to drying to enhance mass transfer and improve product quality (Ding et al. 2026). These pretreatments can modify internal tissue structure and facilitate moisture migration, thereby improving drying kinetics and final product quality (Şimsek and Süfer 2021; Nowacka et al. 2024; Jiang et al. 2024; Wang et al. 2026).

Microwave pretreatment is known for its rapid volumetric heating and high energy efficiency, which can significantly accelerate moisture removal. Ultrasound pretreatment induces cavitation effects that create microchannels in plant tissues, enhancing water diffusion (Zhang et al. 2026; Wang et al. 2026). Infrared pretreatment provides rapid surface heating, improves energy efficiency, and shortens drying time while maintaining product quality (Sakare et al. 2020). For instance, Shewale and Hebbar (2017) reported that infrared pretreatment reduced drying time by up to 23% in apple drying systems. Freezing pretreatment is another effective non‐thermal technique that enhances drying rate by forming ice crystals that disrupt cell structure and increase porosity, although it may also influence final product quality depending on crystal size (Vallespir, Cárcel, et al. 2018; Ciurzyńska et al. 2021).

Developing innovative processing techniques to create nutritious and healthier snack options from vegetable sources is important because of the increase in snack consumption in today's diet and health concerns. Despite the growing interest in beetroot processing, studies evaluating the combined effect of different pretreatments with vacuum drying on drying kinetics and multi‐structural quality attributes of beetroot snacks remain limited. Comprehensive evaluations integrating drying behavior, bioactive compound retention, color microstructural characteristics, rehydration, texture, thermal properties, and functional group analysis are still scarce in the literature. Therefore, the aim of this study was to investigate the effect of different pretreatments (microwave, ultrasound, infrared, and freezing) combined with vacuum drying on the drying kinetics and quality characteristics of red beetroot snacks. In addition, the microstructural, textural, thermal properties, and functional groups of the dried samples were evaluated to provide a comprehensive characterization of the final products.

## 2 Material and Method

### 2.1 Material

Fresh red beetroots were purchased from a producer in Kadirli district of Osmaniye, Türkiye. The red beetroots were cleaned with tap water and peeled with stainless steel knives. Then a vegetable slicer (Status 115010, Slovenia) was used to cut them into 3‐mm thick slices. The samples had an initial weight of 13.16 ± 0.40 g and a diameter of 70.49 ± 0.96 mm. Prior to the trials, moisture content of the red beetroots was determined as 92.64% ± 0.40%.

Prior to drying, red beetroots were subjected to no pretreatment (NP) as control, freezing pretreatment (FP), infrared pretreatment (IP), microwave pretreatment (MWP) and ultrasound pretreatment (USP).

### 2.2 Pretreatments

1. *Freezing pretreatment*: red beetroots were put in a single layer inside Ziploc bags and stored at least 24 h at −18°C in a freezer (Arçelik) until the drying trials. Frozen samples were put quickly into preheated vacuum dryer without thawing.
2. *İnfrared pretreatment*: infrared pretreatment was carried out for 30 min at 50°C using an infrared dryer (Adam, PMB53, USA) with a 400 W, 230 VAC halogen light. The average moisture ratio of the samples was 0.56 ± 0.025 before drying. Weight of samples was recorded in 5 min.
3. *Microwave pretreatment*: microwave pretreatment was carried out at 120 W for 20 min using a household microwave oven (Arçelik, MD 574, Türkiye, 26.2 × 45.2 × 32.5 cm) operating in a discontinuous system and maximum power of 700 W, having a capacity of 17 L. Preliminary experimental findings indicated that 120 W was the ideal microwave power to achieve the optimal drying properties for sample slices without resulting in product burning and color loss. The average moisture ratio of the samples was 0.42 ± 0.069 prior to vacuum drying. Weighing intervals for the samples were set at 5 min.
4. *Ultrasound pretreatment*: samples were placed in a 250 mL beaker filled with water as the medium. The water to raw sample ratio was 1:4 (w/w). Ultrasound treatments were conducted in an ultrasonic bath (Isolab, Germany) at 360 W and a frequency of 40 kHz for 10 min at 25°C. Then, samples were kindly blotted using filter paper to get rid of excess water. Additionally, solid loss and weight gain were computed according to Wang et al. (2019). Solid loss (%) and weight gain (%) were determined as 2.39 ± 0.03 and 1.87 ± 0.04, respectively.

### 2.3 Drying Treatment

**FIGURE 1:**
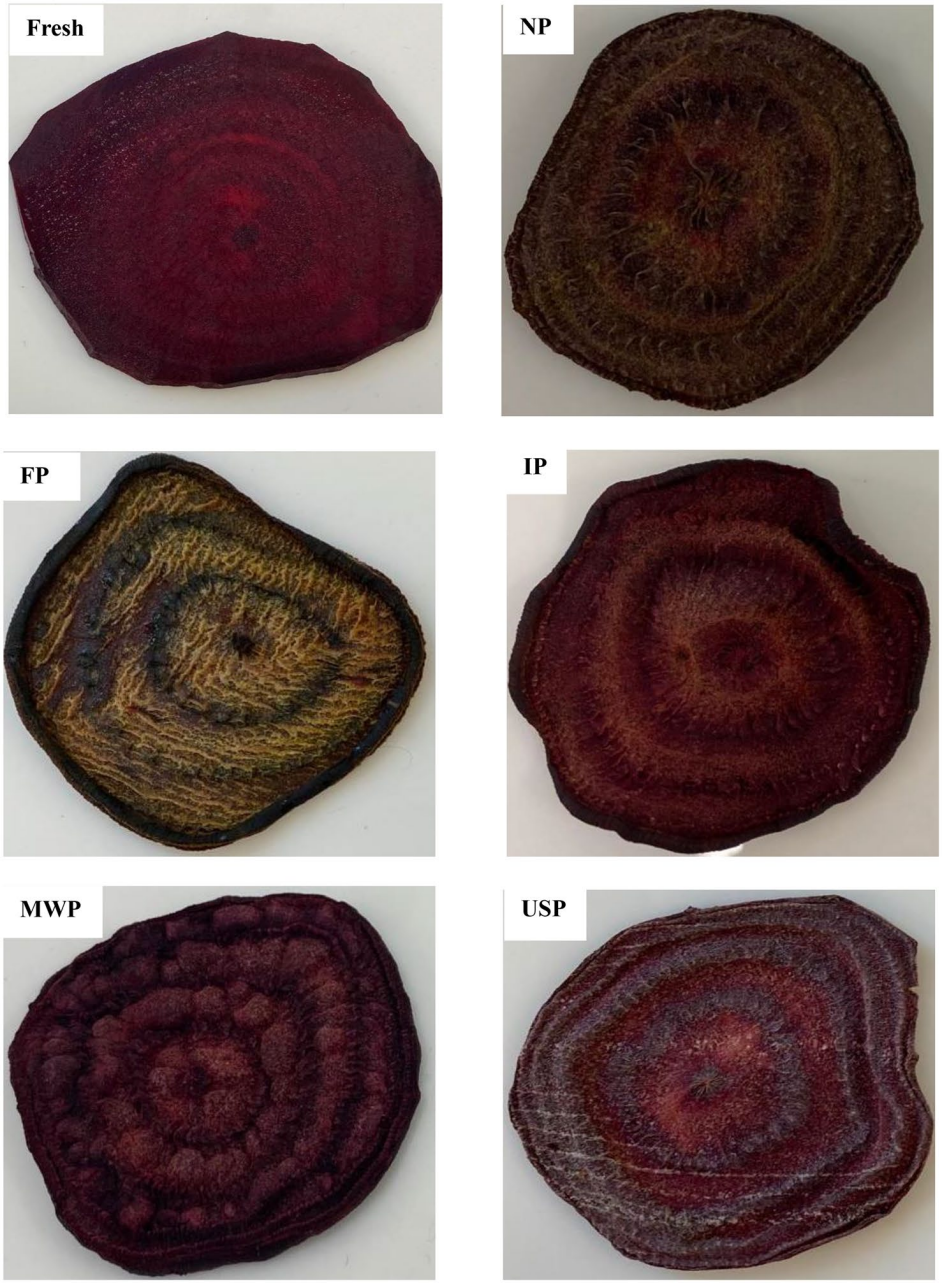
Images of red beetroot samples.

Vacuum drying was conducted at temperature (50°C) and absolute pressure (0.05 MPa), in a vacuum oven (BIOBASE, BOV30V, China). This is a relatively mild vacuum. During the drying, data was recorded every 30 min using a digital balance (Adam, Nimbus, USA). Drying experiments were completed when sample weights remained stable, indicating that no further significant moisture loss occurred. Furthermore, the moisture contents of the dried samples were 8.64 ± 0.30, 8.25 ± 0.23, 8.32 ± 0.62, 8.46 ± 0.28, and 8.05% ± 0.34% for NP, FP, IP, MWP, and USP, respectively. All pretreatments and drying trials were conducted in triplicate. Images of red beetroot samples were given in Figure 1.

### 2.4 Mathematical Modeling of Drying Curves

Eleven different thin layer drying models (Table S1) from the literature were fitted using all drying data by OriginPro 2019 (Origin‐Lab, USA) software. The dimensionless moisture ratio (MR) was calculated using the formula below (Pei et al. 2023).

(1) $$\mathrm{MR}=\frac{M-\mathrm{Me}}{\mathrm{Mo}-\mathrm{Me}}$$

where *M* is the moisture content at any time (kg_water_/kg_dm_); *M*_*o*_ is the initial moisture amount (kg_water_/kg_dm_); *M*_*e*_ is the equilibrium moisture level (kg_water_/kg_dry matter_) and is accepted zero. Therefore, Equation (1) was converted to *M*/*M*_0_ (Özkan‐Karabacak et al. 2020; Mella et al. 2022).

**TABLE 1:** Statistical results of suitable models for drying process.

Samples	Models	*R* ^2^	*χ* ^2^	RMSE	*D*_eff_ (m^2^/s)	*R* ^2^
NP	Page	0.9872	0.0010	0.03023	8.82 × 10^−9^	0.9836
	Modified page	Not converged				
	Approximation of diffusion	0.9881	0.0010	0.02914		
	**Sigmoid**	**0.9949**	**0.0005**	**0.01835**		
	Cubic	0.9944	0.0005	0.01997		
	Parabolic	0.9899	0.0009	0.02685		
	Vega Lemus	0.9792	0.0017	0.03849		
	Rational	0.9907	0.0008	0.02569		
	Vega Galvez 1	0.9332	0.0053	0.08301		
	Vega Galvez 2	0.9826	0.0014	0.03517		
FP	Page	0.9895	0.0010	0.02843	7.55 × 10^−9^	0.9833
	Modified page	Not converged				
	Approximation of diffusion	Not converged				
	Sigmoid	0.9946	0.0006	0.02045		
	**Cubic**	**0.9997**	**0.0000**	**0.00501**		
	Parabolic	0.9997	0.0000	0.00509		
	Vega Lemus	0.9754	0.0023	0.04354		
	Rational	0.9997	0.0000	0.00504		
	Vega Galvez 1	0.9973	0.0002	0.01440		
	Vega Galvez 2	0.9402	0.0055	0.06792		
IP	Page	0.9818	0.0017	0.03808	1.26 × 10^−8^	0.9748
	Modified page	Not converged				
	**Approximation of diffusion**	**0.9903**	**0.0010**	**0.02468**		
	Sigmoid	0.9590	0.0044	0.05709		
	Cubic	0.9659	0.0036	0.05208		
	Parabolic	0.9315	0.0067	0.07381		
	Vega Lemus	0.9157	0.0077	0.08186		
	Rational	0.9787	0.0023	0.04114		
	Vega Galvez 1	0.8663	0.0122	0.10310		
	Vega Galvez 2	0.9457	0.0049	0.06571		
MWP	Page	0.9068	0.0109	0.09324	2.52 × 10^−8^	0.8957
	Modified page	0.8489	0.0176	0.11867		
	Approximation of diffusion	0.8489	0.0201	0.11867		
	**Sigmoid**	**0.9878**	**0.0019**	**0.03367**		
	Cubic	0.8800	0.0187	0.10578		
	Parabolic	0.7589	0.0321	0.14990		
	Vega Lemus	0.7482	0.0293	0.15321		
	Rational	0.9771	0.0036	0.04623		
	Vega Galvez 1	0.5754	0.0495	0.19894		
	Vega Galvez 2	0.8490	0.0176	0.11864		
USP	Page	0.9957	0.0003	0.01575	6.30 × 10^−9^	0.9793
	Modified page	0.9667	0.0022	0.04388		
	Approximation of diffusion	0.9958	0.0003	0.01561		
	Sigmoid	0.9984	0.0001	0.03702		
	**Cubic**	**0.9992**	**0.0001**	**0.00697**		
	Parabolic	0.9991	0.0001	0.00734		
	Vega Lemus	0.9944	0.0004	0.01805		
	Rational	0.9992	0.0001	0.00696		
	Vega Galvez 1	0.9986	0.0001	0.07424		
	Vega Galvez 2	0.9763	0.0016	0.03702		

*Note:* Each pretreatment and drying condition was performed in three independent processing batches (*n* = 3). Bold values indicate the best‐fitting model for each sample, corresponding to the highest coefficient of determination (R²) and the lowest chi‐square (χ²) and root mean square error (RMSE) values.

**TABLE 2:** The constants and coefficients of the best models.

Sample	Models	The best model constants and coefficients				
		*k* (min^−1^)	*a*	*b*	*c*	*d*
NP	Sigmoid	−0.0105	0.1561	0.9502	158.3386	
FP	Cubic		1.0018	−0.0022	0.0000	0.0000
IP	Approximation of diffusion	0.0596	0.3390	0.0892		
MWP	Sigmoid	−0.2226	0.1731	0.9247	10.0017	
USP	Cubic		0.9983	−0.0018	0.0000	0.0000

The most suitable model for each drying was selected the highest *R* ^2^ (Equation 2) root mean square error (RMSE) (Equation 3) and the lowest chi square (*χ* ^2^) (Equation 4).

(2) $$R^{2}=1-\frac{\sum_{i=1}^{N}{\left({\mathrm{MR}}_{\mathrm{pre},i}-{\mathrm{MR}}_{\exp,i}\right)}^{2}}{\sum_{i=1}^{N}{\left({\mathrm{MR}}_{\mathrm{pre}}-{\mathrm{MR}}_{\exp,i}\right)}^{2}}$$

(3) $$\text{RMSE}={\left[\frac{1}{N}\sum_{İ=1}^{N}{\left({\mathrm{MR}}_{\exp,i}-{\mathrm{MR}}_{\mathrm{pre},i}\right)}^{2}\right]}^{1/2}$$

(4) $$\chi^{2}=\frac{\sum_{İ=1}^{N}{\left({\mathrm{MR}}_{\exp,i}-{\mathrm{MR}}_{\mathrm{pre},i}\right)}^{2}}{N-z}$$

where MR_exp,*i*_ is experimental MR at *i*; MR_pre,*i*_ is the predicted MR at *i*; *N* is the number of experimental data and *z* is the number of constants in model.

### 2.5 Effective Moisture Diffusivity

The effective moisture diffusivity (*D*_eff_) was computed based on Fick's second law. Some assumptions were accepted as (a) uniform distribution of temperature and moisture, (b) symmetric mass transfer, (c) constant diffusion coefficient, (d) infinite slab geometry (Özkan‐Karabacak et al. 2020; Nistor et al. 2017; Mella et al. 2022). For longer drying times (*n* = 1), Equation (6) was obtained from Equation (5).

(5) $$\mathrm{MR}=\frac{8}{\pi^{2}}\;\sum_{n=1}^{\infty }\frac{4}{{\left(2n-1\right)}^{2}}\;\exp\left(-\frac{\;{\left(2n-1\right)}^{2}\pi^{2}\;D_{\mathrm{eff}}\;t}{4L^{2}}\right)$$

(6) $$\mathrm{MR}=\frac{8}{\pi^{2}}\exp\left(-\frac{\;\pi^{2}\;D_{\mathrm{eff}}\;t}{4L^{2}}\right)$$

*D*_eff_ is calculated as the slope of a straight‐line plotting ln MR versus time (*t*):

(7) $$D_{\mathrm{eff}}=\frac{\text{slope}\;4L^{2}\;}{\pi^{2}}$$

### 2.6 Determination of Bioactive Compounds and Antioxidant Activity

Bioactive compounds extraction was carried out using a procedure modified from Da Silva et al. (2023). One gram powder and 25 mL of 50% ethanol solution were mixed in centrifuge tube (50 mL). The mixture vortexed vigorously for 1 min and put in the ultrasonic bath for 30 min at 25°C. Then, the mixture was centrifuged for 20 min at 4430 g, and the extracts were collected and stored at −20°C until analyses.

#### 2.6.1 Betalain Content With Spectrophotometric Analysis

A spectrophotometric approach based on absorbance measurements at 480 nm for betaxanthins and 538 nm for betacyanins was used to calculate the betalain content of the extracts. The extraction was performed according to a solvent extraction procedure with slight modifications. Briefly, 1 g of powder sample was mixed with 25 mL of 50% (v/v) ethanol solution and subjected to extraction in an ultrasonic bath at 25°C for 30 min. Following extraction, the samples were centrifuged at 4430 g for 10 min. The supernatants were then collected and filtered. The extracts were diluted with 50% ethanol solution (1:10). Equation (8) was utilized to compute the betalain content, which was expressed as mg/kg and was derived from the sum of betacyanins and betaxanthins (Seremet (Ceclu) et al. 2020).

(8) $$\text{Betalain}\;\left(\frac{\mathrm{mg}}{\mathrm{kg}}\right)=\frac{\left(A\times \mathrm{DF}\times \mathrm{MW}\times 1000\right)\;}{\left(\varepsilon \times 1\right)}$$

where *A* is the value of the absorbance, *L* = 1, is the path length, *ε* is the molar extinction coefficient, DF is the coefficient of dilution factor and MW is the molecular weight. Molar extinction coefficients in water and molecular weights were used to quantify betaxanthin and betacyanin. For betacyanin, *ε* = 60,000 L/mol cm and MW = 550 g/mol, and for betaxanthin *ε* = 48,000 L/mol cm and MW = 339 g/mol.

#### 2.6.2 Betalain Compounds Identification With HPLC Analysis

*Betalain* compounds were determined using an HPLC method, which was modified by Sawicki et al. (2016). HPLC (Shimadzu LC‐20AT Japan) system consisted of a quaternary pump, a column temperature control oven (CTO‐10AS), an auto‐sampler unit (SIL‐20A), a degasser module (DGU20A5), and a photodiode array detector (PDA, SPD‐M20A) set at 480 and 535 nm. 20 μL supernatant or standard were injected into a column C18 (Waters, 250 × 4.6 mm, 5 μm pore diameter) at 30°C. The flow rate was adjusted to 1 mL/min with water‐formic acid (95:5, v/v, phase A) and acetonitrile‐phase A (80:20, v/v, phase‐B) as mobile phase. Based on preliminary experiments, the gradient elution was A 100% + 0% B in 0 min, 65% A + 35% B in 70 min, 10% A + 90% B in 72 min and 100% A + 0% B in 77 min. Betanin and isobetanin were quantified by comparison to an external standard of betanin (Sigma‐Aldrich Co.). The calibration curve (*R* ^2^ = 0.998) was obtained using the external standard of betanin (mg/kg) (Sigma‐Aldrich Co.). Vulgaxanthin I was tentatively identified by means of a comparison of their retention time *λ*_max_ value (Koubaier et al. 2014; Sawicki et al. 2016; Wiczkowski et al. 2018).

#### 2.6.3 Total Phenolic Content (TPC) and Antioxidant Activity

TPC was determined using the Folin–Ciocalteu method (Abdullakasim et al. 2007). The utilized standard was gallic acid (Sigma‐Aldrich, USA), and the TPC was reported as milligrams of gallic acid equivalents (mg GAE/kg). Antioxidant Activity (AA) of the extract was determined according to DPPH analyses (Klimczak et al. 2007). Absorbance of the sample was measured at 515 nm. Results were expressed as % inhibition of DPPH. Equation (9)

(9) $$\text{DPPH}\left(\%\right)=\frac{A_{\text{control}}-A_{\text{sample}}}{A_{\text{control}}}\times 100$$

### 2.7 Color Analysis

The color of the dried samples was measured using a color meter (Konica Minolta CR‐400, Japan). Lightness (L*), redness (a*), yellowness (b*) was measured via reflectance equipment. The total color difference (Δ*E*), chroma (C*), and Hue angle (*h*°) values were calculated according to Equations (10), (11), and (12), respectively.

(10) $$\Delta E=\sqrt{{\left(L^{*}-L_{0}^{*}\right)}^{2}+{\left(a^{*}-a_{0}^{*}\right)}^{2}+{\left(b^{*}-b_{0}^{*}\right)}^{2}}$$

(11) $$C^{*}=\sqrt{\left(a^{*2}+b^{*2}\right)}$$

(12) $$h^{\circ }=\arctan\left(\frac{b^{*}}{a^{*}}\right)$$

$L_{0}^{*}$, $a_{0}^{*}$, and $b_{0}^{*}$ show the color values of fresh red beetroot slices.

### 2.8 Browning Index

Browning index (BI) was determined spectrophotometrically according to the method of Maity et al. (2016) with minor modifications. Briefly, 1 g of the dried beetroot powder was extracted with 25 mL of 67% (v/v) ethanol for 1 h at room temperature. The extract was subsequently filtered, and the absorbance was measured at 420 nm using a UV–Vis spectrophotometer. Ethanol solution (67% (v/v)) was used as the blank.

### 2.9 Rehydration Ratio

2 g of dried red beetroot slices were placed in 150 mL of distilled water contained in 250 mL glass beakers. The experiments were conducted at room temperature (25°C ± 2°C) Following immersion, the samples were removed at 60 min intervals, drained to eliminate excess surface water, and weighed until no further change in mass was observed. The rehydration experiments were carried out in triplicate, and the rehydration ratio was determined from the recorded mass changes (İnan‐Çınkır et al. 2024).

### 2.10 Water Activity (*a*_*w*_)

Water activity values were determined using water activity measuring device (Novasina brand LabMASTER, Switzerland). Before analysis, the device was calibrated with salts with different water activity.

### 2.11 Texture Profile Analysis

Hardness values were obtained by a texture analyzer (Brookfield, CT3, USA) which used software called Texturepro CT V1.4 Build 17. Texture profile analysis (TPA) was performed according to modified method by Liu et al. (2022). Pre‐test and test speeds were 1 mm/s, and trigger load was 0.05 N. The device load was 4.500 g and a cylinder probe (TA5, D = 12.7 mm, L = 35 mm) pressurized to samples. Each dried sample was subjected to the TPA at least eight times.

### 2.12 Scanning Electron Microscope (SEM) Analysis

A scanning electron microscope (SEM) (FEI Quanta FEG 650, USA) was used to analyze the morphologies of the dried samples following gold coating. All SEM tests were conducted between 10 and 20 kV.

### 2.13 ATR‐FTIR Analysis

Functional groups of dried samples were determined by an attenuated total reflectance Fourier transform infrared (ATR‐FTIR) spectrometer (Perkin Elmer Spectrum 65, USA). The scan range was between 4000 and 800 cm^−1^ at 4 cm^−1^. The spectrum of each sample was obtained after an average of 12 scans (Kuznietsova et al. 2020).

### 2.14 Differential Scanning Calorimetry (DSC)

The thermal behavior was detected by differential scanning calorimetry (DSC) (Mettler Toledo, DSC3, Switzerland). The samples were heated from 25°C to 200°C at a rate of 10°C/min under a constant flow rate of nitrogen gas of 40 mL/min. The DSC instrument's software was used to calculate all data. The DSC curve was used to determine the onset (*T*_*o*_), peak (*T*_*p*_), and endset (*T*_*e*_) temperatures, as well as the enthalpy change (Δ*H*) (Kaur et al. 2021).

### 2.15 Statistical Analysis

All analyses were performed in triplicate. The experimental data was expressed as mean ± standard deviation. The obtained results were subjected to variance analysis using the IBM SPSS V20.0 package program, and significant differences were determined according to the Duncan multiple comparison test (*p* < 0.05).

## 3 Results and Discussion

### 3.1 Drying

**FIGURE 2:**
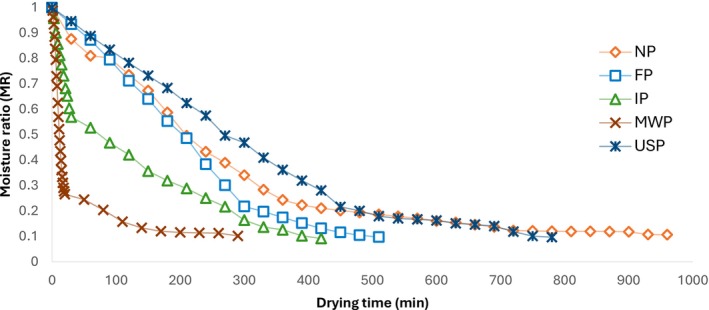
The drying curves of different pretreated red beetroot samples.

The change in MR with drying time for NP and pretreated samples was displayed in Figure 2. The curves showed a rapid loss of water according to the pretreatment process, followed by a reduction in moisture. The moisture ratio declined in the following order: MWP > IP > FP > USP > NP. The drying times were 290, 420, 510, 750 and 900 min for MWP, IP, FP, USP and NP respectively. Total processing time (pretreatment+drying) was 310 min (20 + 290) min, 450 min (30 + 420), 1950 min (1440 + 510) and 760 min (10 + 750) for MWP, IP, FP and USP respectively. Compared with NP, the overall drying time was reduced by 67.78%, 53.33%, 43.33%, and 16.67% for MWP, IP, FP, and USP, respectively. According to the data, heat and mass transfer occur more quickly with microwave pretreatment. Because heat is produced within the tissue as opposed to being transported. It results in a greater vapor pressure differential between the product's surface and inside compared to other pretreatment approaches (Nistor et al. 2017; Salehi et al. 2023). These findings were consistent with earlier studies that employed microwaves as a pretreatment before vacuum drying orange (Özkan‐Karabacak et al. 2020), freeze drying pumpkin (Wodajo Bekele and Admassu 2022), hot‐drying carrot (Salehi et al. 2023).

The MR data of NP and all pretreatment (Table 1) were subjected to the thin layer drying models listed in Table S1. Bold values indicate the best‐fitting model for each sample, corresponding to the highest coefficient of determination (R²) and the lowest chi‐square (χ²) and root mean square error (RMSE) values. The constants and coefficients of the best models were given Table 2. To represent the thin layer drying of red beetroots, the Sigmoid (*R* ^2^ = 0.9949), Cubic (*R* ^2^ = 0.9997), Approximation of diffusion (*R* ^2^ = 0.9903), Sigmoid (*R* ^2^ = 0.9878) and Cubic (*R* ^2^ = 0.9992) models were selected as best models for the NP, FP, IP, MWP, and USP, respectively (Table 1). As far as known, the suitability of thin layer models used in this study were not tested for drying kinetic of red beetroots, except for page and modified page in the literature. Sigmoid model was found to be the best fit model for watermelon radish (Çınkır and Süfer 2020) and white sweet cherries (Şimsek and Süfer 2021).

The use of different best‐fit models is not considered merely an artifact of curve fitting but is likely related to the modifications in moisture transport behavior induced by each pretreatment. Pretreatments such as microwave, ultrasound, infrared, and freezing can alter the internal structure, porosity, and moisture migration pathways of beetroot tissues, leading to differences in drying kinetics (Jiang et al. 2025). Consequently, different mathematical models may better describe the drying behavior of samples subjected to different pretreatments. Nevertheless, all selected models provided excellent goodness‐of‐fit statistics and were used primarily to accurately represent the experimental drying data.

D_eff_ values were displayed in Table 1. While the minimum *D*_eff_ value (8.82 × 10^−9^ m^2^/s) was obtained in NP, maximum *D*_eff_ value (2.52 × 10^−8^ m^2^/s) corresponded to MWP. These values were properly within the typical range of 10^−11^ to 10^−6^ m^2^/s (Ahmed et al. 2023). The moisture diffusivities of all pretreatments were higher compared to that of NP. Thus, drying times shortened, and drying rates increased. The microwave induces thermal changes in the membrane components of the cell structure, which increases porosity. This results in improved water diffusivity in the dried vegetable product (Nistor et al. 2017). Mella et al. (2022) determined that *D*_eff_ values of vacuum dried red beetroot (40°C–80°C) were between 0.650 × 10^−10^ and 3.566 × 10^−10^ m^2^/s. These values were low compared to NP. This may be related that the transfer of mass and heat in vacuum operations can be challenging due to the reduced effectiveness of heat convection at low pressure (Mella et al. 2022). Ahmed et al. (2023) found that the effective moisture diffusivity of microwave‐dried beetroots changed from 1.78 × 10^−8^ to 6.6 × 10^−8^ m^2^/s for 300–800 W.

**TABLE 3:** Bioactive compounds and antioxidant activity of dried red beetroot samples.

Sample	Betaxanthin (mg/kg)	Betacyanin (mg/kg)	Total betalain (mg/kg)	TPC (mg/kg)	Antioxidant activity (%)
NP	2504.10 ± 10.39^d^	3635.16 ± 9.36^d^	6139.25 ± 4.60^d^	5699.80 ± 3.32^d^	59.38 ± 0.11^a^
FP	1955.87 ± 27.01^e^	1206.56 ± 25.00^e^	3162.43 ± 3.65^e^	4967.38 ± 0.00^e^	36.82 ± 0.00^e^
IP	3028.49 ± 7.61^c^	4793.59 ± 7.30^c^	7822.08 ± 8.95^c^	5826.76 ± 3.90^c^	58.69 ± 0.02^b^
MWP	3293.33 ± 6.07^b^	5063.44 ± 6.61^b^	8356.77 ± 9.24^b^	6402.93 ± 4.10^b^	54.75 ± 0.05^d^
USP	3517.12 ± 26.80^a^	4986.09 ± 27.75^a^	8503.22 ± 4.92^a^	6158.79 ± 3.12^a^	57.55 ± 0.00^c^

*Note:* Values are presented as mean ± standard deviation. Each pretreatment and drying condition was performed in three independent processing batches (*n* = 3), and each analytical measurement was performed in triplicate for each independent batch (3 replicates per batch). Different letters in the same column indicate significant differences (*p* < 0.05).

Abbreviations: FP, freezing pretreatment; IP, infrared pretreatment; MWP, microwave pretreatment; NP, non‐pretreatment; TPC, total phenolic content; USP, ultrasound pretreatment.

### 3.2 Effect of Different Pretreatments on Bioactive Compounds and Antioxidant Activity of Vacuum‐Dried Red Beetroots

Betaxanthin, betacyanin and total betalain for all samples were determined spectrophotometrically (Table 3). Betaxanthin, betacyanin and total betalain values for extracts of all samples were 1955.87–3517.12, 1206.56–4986.09 and 3162.43–8503.22 mg/kg, respectively. The difference between betaxanthin, betacyanin and total betalain values was statistically significant among themselves (*p* < 0.05). The highest betalain values were obtained with USP followed by MWP. USP and MWP as a potential green technique preserved more betalains in the red beetroots. Several studies emphasized the positive effects of ultrasound and microwave on drying. Seremet (Ceclu) et al. (2020) determined that high betalains retention was obtained with hybrid dried beetroot with microwave pretreatment. Liu et al. (2021) reported that betacyanin and betaxanthin values were 3.38–4.65 and 2.52–3.34 mg/g in vacuum‐microwave dried beetroot. Additionally, Szadzińska et al. (2020) found that the highest betalains retention was obtained in convective‐ultrasound hybrid drying for red beetroot cubes.

FP had lowest betalain values. It seems that freezing pretreatment had an impact on the structure of the beetroot cells and increased the reaction between various chemicals to produce new free compounds (Vallespir, Cárcel, et al. 2018). Similarly, Vallespir, Cárcel, et al. (2018) reported that betalains of red beetroot showed high declines in convective drying (assisted or non‐assisted by ultrasound) with freezing pretreatment. Liu et al. (2024) determined that the betacyanin and betaxanthin contents changed 2.78–4.38 and 2.37–3.12 mg/g of freeze–thaw pretreated beetroots obtained using various drying.

Betacyanin stability was higher than betataxanthin for all samples except that FP in this study. The results were consistent with the literature (Sanchez‐Gonzalez et al. 2013; Nutter et al. 2021). It was highlighted that the glycosylation of betacyanin results in an increased redox potential, and thus less reactivity with molecular oxygen is primarily responsible for this stability (Nutter et al. 2021).

According to HPLC analyses, three main compounds in extracts of all samples were determined as follows: vulgaxanthin I as betaxanthin at 480 nm and betanin and isobetanin as two betacyanins at 535 nm, respectively. A representative chromatogram of betalain compounds was given in Figure S1. Vulgaxanthin I, betanin and isobetanin were 2539.89–3678.18, 308.27–4048.94 and 194.22–1115.95 mg/kg, respectively (Table 4). The difference between values was statistically significant among themselves (*p* < 0.05). The studies stated that the predominant betalain components of red beetroots were betanin, isobetanin, and vulgaxanthin I (Koubaier et al. 2014; Fernando et al. 2022; Sawicki et al. 2016; Da Silva et al. 2023). The identification of the compounds was performed by external standards, interpreting the obtained fragmentation spectrum or by comparing their retention time and *λ*_max_ value with the previous research. Isobetanin concentrations were expressed as betanin equivalents and should be considered semi‐quantitative. In addition, vulgaxanthin I was tentatively identified based on retention time and UV–Vis spectral characteristics without the use of a pure reference standard; therefore, its concentration should not be considered an absolute quantification.

**TABLE 4:** Main betalain compounds of dried red beetroot samples.

Sample	Vulgaxanthin I (mg/kg)	Betanin (mg/kg)	İsobetanin (mg/kg)
NP	2862.16 ± 4.59^d^	2413.77 ± 14.95^d^	1023.97 ± 4.98^c^
FP	2539.89 ± 30.65^e^	308.27 ± 10.83^e^	194.22 ± 22.94^e^
IP	2858.83 ± 18.95^c^	3383.97 ± 19.82^b^	848.34 ± 3.14^d^
MWP	2938.24 ± 9.23^b^	3351.81 ± 7.98^c^	1115.95 ± 6.31^a^
USP	3678.18 ± 14.93^a^	4048.94 ± 11.16^a^	1077.05 ± 24.07^b^

*Note:* Values are presented as mean ± standard deviation. Each pretreatment and drying condition was performed in three independent processing batches (*n* = 3), and each analytical measurement was performed in triplicate for each independent batch (3 replicates per batch). Different letters in the same column indicate significant differences (*p* < 0.05).

HPLC analysis results were partially consistent with spectrophotometric analysis. Total betalain concentration was computed to formulations with known extinction coefficients for betacyanins and betaxanthins (Nemzer et al. 2011). The findings of this investigation, particularly for the yellow components, determined as a rough estimate because different betacyanin derivatives with unknown extinction coefficients are present. Similar results were obtained by Nemzer et al. (2011).

TPC values varied from 4967.38 to 6402.93 mg/kg (Table 3). The difference between TPC values was statistically significant (*p* < 0.05). The highest TPC value was obtained with MWP following USP, IP, NP and FP. This can be explained that MWP samples were obtained shorter drying times compared to other samples and microwave radiation causes phenolic compounds to be released from the food matrix (Salehi et al. 2023; Liu et al. 2024). Liu et al. (2024) reported that the highest total phenolic content of dried beetroots was obtained with microwave‐vacuum drying. Souza da Silva et al. (2019) reported that the ultrasound pretreatment before vacuum drying provided greater retention for the phenolic compounds of nectarine. Vallespir, Cárcel, et al. (2018) reported that dried red beetroot had low TPC values in convective drying with freezing pretreatment. Liu et al. (2022) reported that TPC value of dried beetroot was 9.26 mg GAE/100 g in vacuum drying followed by microwave drying.

Antioxidant activity (DPPH) values were 36.82%–59.38% for all samples (Table 3). The highest (59.38%) and lowest (36.82%) DPPH values were obtained with NP and FP, respectively. The phenolic compounds and betalains of the extracts were linked to the DPPH radical scavenging activities. The difference between DPPH values was statistically significant (*p* < 0.05). Betalains and polyphenols both exhibit high radical scavenging properties (Fernando et al. 2022). These values were close to the values in this study. Vallespir, Cárcel, et al. (2018) reported that antioxidant activity of dried red beetroot showed high declines (32%–81%) in convective drying (assisted or non‐assisted by ultrasound) with freezing pretreatment.

For the highest antioxidant activity of NP, a decrease in phenolic compounds and betalain content and an increase in antioxidant activity may be caused by the intermediate oxidation state of polyphenols, the generation of Maillard reaction products, and an increase in reducing sugar. A non‐enzymatic browning process triggered by heat treatment resulted in partially oxidized polyphenols. The oxidized polyphenols have greater antioxidant potential for antioxidant activity than non‐oxidized phenols (Manzocco et al. 2000). This result was consistent with BI values (Table 5). Furthermore, high browning index observed in the NP samples may be associated with the relatively mild vacuum conditions and longer drying duration. These conditions likely favored the progression of non‐enzymatic browning reactions.

**TABLE 5:** Color values of red beetroot samples.

Sample	L*	a*	b*	Δ*E*	C*	*h*°	BI
Fresh	21.66 ± 0.56^f^	24.13 ± 0.36^e^	9.15 ± 0.17^f^	—	25.81 ± 0.32^f^	20.77 ± 0.53^c^	—
NP	23.33 ± 0.16^e^	31.16 ± 0.50^d^	11.44 ± 0.45^e^	15.67 ± 0.49^d^	33.27 ± 0.53^d^	26.61 ± 0.34^b^	0.43 ± 0.05^a^
FP	31.16 ± 0.62^a^	6.37 ± 0.30^f^	26.39 ± 0.83^a^	26.52 ± 0.53^a^	27.62 ± 0.23^e^	76.40 ± 0.88^a^	0.47 ± 0.02^b^
IP	25.37 ± 0.27^c^	34.18 ± 0.50^c^	17.28 ± 0.46^b^	13.76 ± 0.88^c^	38.31 ± 0.64^c^	19.82 ± 0.25^d^	0.55 ± 0.04^b^
MWP	24.25 ± 0.37^d^	37.32 ± 0.82^b^	12.18 ± 0.67^d^	13.41 ± 0.64^c^	39.21 ± 0.82^b^	17.74 ± 0.86^e^	0.27 ± 0.01^c^
USP	29.47 ± 0.20^b^	41.18 ± 0.21^a^	14.34 ± 0.11^c^	19.46 ± 0.19^b^	43.61 ± 0.22^a^	19.19 ± 0.09^d^	0.23 ± 0.02^d^

*Note:* Values are presented as mean ± standard deviation. Each pretreatment and drying condition was performed in three independent processing batches (*n* = 3), and each analytical measurement was performed in triplicate for each independent batch (3 replicates per batch). Different letters in the same column indicate significant differences (*p* < 0.05).

Abbreviations: Δ*E*, total color difference; BI, browning index; C*, chroma; *h*°, Hue angle.

### 3.3 Effect of Different Pretreatments on Color, Rehydration Ratio and Water Activity of Red Beetroots

The color measurement (L*a*b*) showed redness and brightness of the dried beetroot samples as well as the intensity of their bright color. The color values were presented in Table 5. L*, a*, b*, Δ*E*, C*, *h*° and BI values were determined as 21.66–31.16, 6.37–41.18, 9.15–26.39, 13.41–26.52, 25.81–43.61, 17.74–76.40 and 0.23–0.55. The differences between color values for all samples were statistically significant (*p* < 0.05). L* is a crucial parameter in the drying process. Because L* is typically the first quality characteristic that customers evaluate when deciding whether to accept a product, low L* levels are primarily linked to non‐enzymatic browning reactions and show a dark color (Salehi et al. 2023). According to L* values, the pretreatments caused an increase in the L* values of dried beetroot samples. The USP sample had the highest a* value which was consistent with the high concentration of betalains (Table 5). Higher a* values indicate more reddish of a sample (Zin and Bánvölgyi 2023). This situation was correlated with betalain values. Similar results were obtained by Seremet (Ceclu) et al. (2020) and Liu et al. (2024) who reported that red beetroots had lighter red color after drying. Nowacka et al. (2024) reported that high L* value and low a* value of beetroots was found in infrared–hot air drying with ultrasound pretreatment.

The FP sample had the highest b* value. The increase in b* values showed shifting of the color toward yellow. Betaxanthins are responsible for yellow color. The betaxanthin content was more stable than betacyanin for FP sample. Under heat treatment and oxygen exposure, betalains can undergo dehydrogenation and decarboxylation processes that result in yellow‐orange color alterations. Neobetanin from degradation products is mostly responsible for these color changes (Zin and Bánvölgyi 2023). Vallespir, Rodríguez, et al. (2018) determined that beetroot samples showed noticeable color changes following the freezing pretreatments and drying process.

The Δ*E* represents the color difference between the dried and fresh samples. Values greater than 3.0 indicate that the sample differs significantly from the fresh sample, implying that all treatments changed the color of the red beetroots (Özkan‐Karabacak et al. 2020). While FP had the highest Δ*E* value, MWP resulted in the lowest value. C* value is an indicator of the color saturation and intensity of red beetroot slices, reflecting the degree of color vividness. Among the drying treatments, the highest color vividness was observed in USP samples. *h*° values (19.19°–76.40°) were positioned in the first quadrant, confirming the predominance of red color in beetroot samples. However, FP samples exhibited the highest *h*° values together with lower C* values, indicating a less vivid and relatively duller color appearance. Freezing pretreatment may cause cellular disruption, leading to pigment degradation or leakage and consequently reducing color intensity (Zhang et al. 2021).

BI values were presented in Table 5. USP displayed lower BI values, while NP, IP and FP (> 0.4) had higher values (*p* < 0.05). Infrared and freezing pretreatments may have contributed to a further increase in Maillard reactions and ascorbic acid degradation, while, in the case of the NP samples, the extended drying duration likely contributed to the progression of these reactions (Tarasevičienė et al. 2024). In addition, the formation of brown pigment caused a decrease in lightness. Among treatments, NP had the lowest L* value.

**FIGURE 3:**
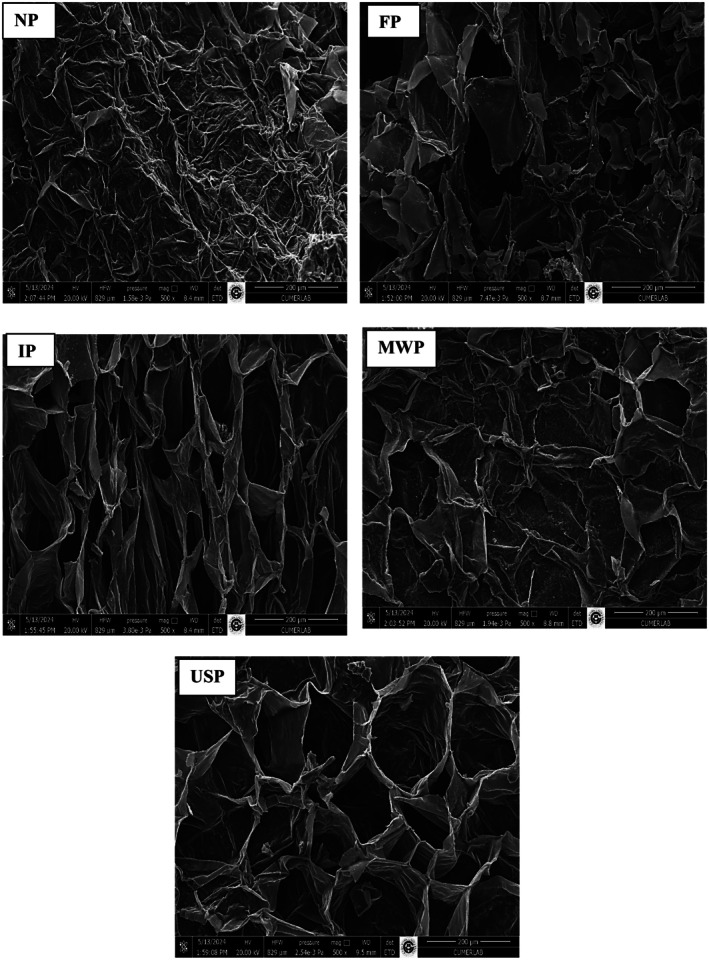
The microstructure (SEM graphs) of dried red beetroot samples.

Rehydration ratio (RR) is defined as the capacity of a dried material to absorb water and restore its original structure and properties when exposed to moisture (İnan‐Çınkır et al. 2024). Rehydration performance is largely governed by the porous structure developed during drying and is therefore strongly affected by processing conditions, pretreatment techniques, and the physicochemical composition of the raw material. Consequently, rehydration behavior serves as an indirect indicator of structural and physicochemical changes occurring throughout the drying process (Özkan Karabacak et al. 2025). The highest RR was observed in USP while the lowest was in NP (Table 6). All pretreatment positively affected RR of the red beetroot slices. These results correlated with the variations in SEM graphs (Figure 3).

**TABLE 6:** Rehydration ratio, water activity (*a*_*w*_) and hardness values of dried red beetroot samples.

Sample	Rehydration ratio (g/g)	*a*_*w*_	Hardness (N)
NP	4.09 ± 0.12^e^	0.338 ± 0.00^c^	38.06 ± 5.34^a^
FP	5.40 ± 0.48^c^	0.313 ± 0.00^b^	5.06 ± 1.05^d^
IP	4.90 ± 0.13^d^	0.310 ± 0.00^d^	3.96 ± 1.99^e^
MWP	5.71 ± 0.06^b^	0.316 ± 0.01^a^	17.97 ± 0.73^b^
USP	6.18 ± 0.24^a^	0.308 ± 0.00^d^	5.75 ± 0.11^c^

*Note:* Values are presented as mean ± standard deviation. Each pretreatment and drying condition was performed in three independent processing batches (*n* = 3), and each analytical measurement was performed in triplicate for each independent batch (3 replicates per batch). Different letters in the same column indicate significant differences (*p* < 0.05).

Water activity (*a*_*w*_) is a crucial parameter for promoting microbial growth and improving food product stability (Malakar et al. 2022). *a*_*w*_ values changed between 0.308 and 0.338 (Table 6). The difference between *a*_*w*_ values was statistically significant (*p* < 0.05). It was still below 0.4, which is known as the important water activity point, at which the rate of enzymatic, hydrolytic, Maillard, lipid oxidation, and microbiological processes increases dramatically (Szadzińska et al. 2020). The lowest aw value was obtained with USP. It was stated that cavitation happens during ultrasound pretreatment. Microscopic channels in tissue are formed that increase convective mass transfer and decrease the diffusion boundary layer (Ciurzyńska et al. 2021). Similar trends about reduction of water activity and high drying rates were reported by Preethi et al. (2020) and Malakar et al. (2022) and Nowacka et al. (2024).

### 3.4 Hardness

Hardness evaluates the sample's textural characteristics. The hardness values of the beetroots were greatly impacted by the various pretreatments. The hardness values were presented in Table 6. The values were determined as 3.96–38.06 N. The differences between hardness values were statistically significant (*p* < 0.05). The pretreatments resulted in a decrease in the hardness values of dried beetroot samples. They likely caused cell wall disintegration, resulting in cellular holes and softened texture (Noshad and Ghasemi 2020; Pei et al. 2023). While NP exhibited the highest hardness value, IP had the lowest value. The texture of IP beetroot samples was soft. For NP, the significant shrinkage of beetroot slices probably led to the development of a harder texture due to the vacuum drying. The results of SEM showed that all samples except NP had porous structures. Because of their porous structure, the hardness values could be reduced. In addition, it is stated that puffing phenomena occurs during the microwave drying process to create a porous texture of product. It reduces shrinkage and density, thus it creates a soft surface (Szadzińska et al. 2020; Ahmed et al. 2023). Szadzińska et al. (2020) reported that hardness values of red beetroot cubes were between 92.3 and 498.1 N.

### 3.5 SEM

SEM images of NP and pretreated samples were given in Figure 3. NP showed structural collapse, thin pore structures, and undetectable cell borders, all of which had an adverse effect on texture (Liu et al. 2024). This was related that the inner temperature of NP was lower than their outside temperature during drying. The resulting harder surface increased microstructural stress (Yao et al. 2020). For IP, infrared radiation provided energy for rapid heating, and thus water vapor created large pores in the samples and enlarged the cellular walls. This could be explained by the high temperature in samples caused by the combining of vacuum and infrared radiation compared to other treatments (Onwude et al. 2019). Similar results were obtained by Onwude et al. (2019) in combined infrared and hot‐air dried sweet potato. Microwave pretreatment provided energy for rapid heating, and thus internal water vapor created large pores in the samples and enlarged the cellular walls. It also led to surface collapse (Nistor et al. 2017; Liu et al. 2022). MWP samples demonstrated a dense layer and large pores. Similar results were obtained by Liu et al. (2022) and Makovic et al. (2025) in microwave assisted vacuum drying of red beetroots.

USP improved pore opening compared to NP (Figure 3). There were less closed or semi‐closed pores and the neighboring cell walls were aligned properly. This might be because both humidity and temperature gradients of samples moved in the same direction, and the drying rate increased resulting in the formation of a porous and open structure (Nowacka and Wedzik 2016; Nowacka et al. 2024; Zang et al. 2024; Jiang et al. 2024). Similar results were reported by Hidangmayum et al. (2023), Zang et al. (2024) and Wang et al. (2026).

FP caused tissue contraction and collapse due to a lack of cell turgor. It severely damaged beetroots' cell structures. Freezing and drying can cause completely cellular damage and abnormal forms, as well as shrinkage (Vallespir, Rodríguez, et al. 2018; Liu et al. 2024). The intercellular spaces were enlarged in FP samples. This phenomenon can be attributed to drying of intracellular fluid and the development of ice crystals in the extracellular space. The initial cell wall structure was broken because of this ice crystal production, creating large holes as previously mentioned by Ando et al. (2019) and Xu et al. (2021). In contrast to these, Vallespir, Rodríguez, et al. (2018) found that untreated dried beetroot had closer pores and microstructure after convective drying.

These microstructural variations correlate with the variations in hardness values as shown in Section 3.4.

### 3.6 ATR‐FTIR

**FIGURE 4:**
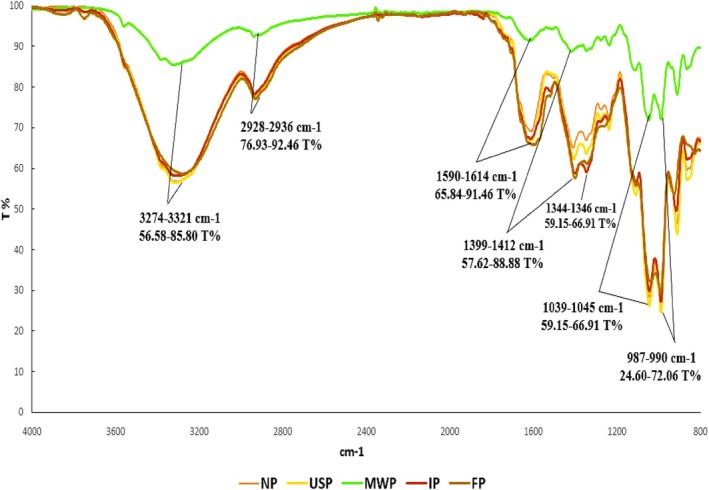
ATR‐FTIR spectra for dried red beetroot samples.

The ATR‐FTIR spectrums and characteristic peaks of NP and all pretreatments were illustrated in Figure 4. Similar functional groups were observed for all samples. The peak variation or weak bands resulted from moisture loss during the pretreatments and drying process. The absorption bands between the ranges of 3274–3321 cm^−1^ showed the presence of hydroxyl groups (O–H stretching of moisture content) (del Amo‐Mateos et al. 2023). The peaks at 2933–2988 cm^−1^ were associated with the C–H stretching vibrations (associated with carboxylic acids such as citric acid) (Mocanu et al. 2020; del Amo‐Mateos et al. 2023). The FP (2988 cm^−1^) increased the peak intensities compared to those of other pretreatments (2933–2936 cm^−1^). The presence of nitrogenous compounds called betalains in the dried beetroot samples was confirmed by the peak that varied from 1590 to 1614.1 cm^−1^ (amide 1 (N–H bending), C=O stretching vibrations) (Singh et al. 2017; Kaur et al. 2021). Absorption spectra between 800 and 1500 cm^−1^ are related to as fingerprint regions because they represent a complicated region with numerous bands that frequently overlap. In addition, the peaks of 1500–1200 cm^−1^ are susceptible to variations in molecular and chemical structures. The absorption spectra varying from 1399 to 1412 cm^−1^ indicated presence of aromatic C–C stretching vibrations, which are observed around 1384–1481 cm^−1^ (Ganga et al. 2023). The peak at 1344–1346 cm^−1^ were associated with O–H and C–H groups (Kaur et al. 2021). The bands appearing from 1107 to 1236 cm^−1^ were exhibited C–O stretching. The bands demonstrated the presence of phenols and alcohol. Spectral range of 1300–1000 cm^−1^ shows an identifiable band for phenols and alcohols. The observed bands at 1039–1045 cm^−1^ correspond to the ether bond found in carbohydrates (Beluhan et al. 2022). The characteristic peak of the C=C stretching vibrations (alkene) were observed in range of 987–990 cm^−1^ (Kaur et al. 2021). The peaks at 864–925 cm^−1^ resulted from C–OH stretching vibration. The intensity of peak at 863–864 cm^−1^ disappeared in FP.

### 3.7 DSC

**FIGURE 5:**
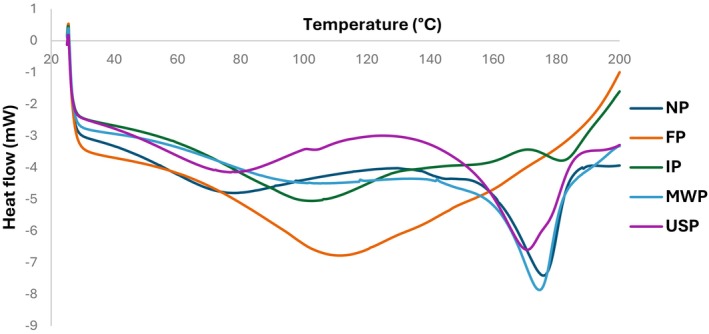
DSC curves of dried red beetroot samples.

DSC thermograms were presented in Figure 5. DSC curves of all samples had two endothermic peaks except for FP. It seemed that pretreatments decreased the size of endothermic peaks. The first endothermic peaks (T_p1_) of 75.35°C, 76.04°C, 94.68°C and 98.99°C resulted in the separation of the free moisture and the breakdown of compounds for NP, USP, MWP and IP, respectively (Table S2). These results were related that betacyanins and betaxanthins have hydrophilic groups that can create hydrogen bonds with water. USP, MWP and IP had more betalain compounds compared to NP, and thus hydrogen bonds increased. The second endothermic peaks (T_p2_) of 175.67°C, 170.38°C, 174.32°C and 182.50°C showed melting points of compounds, as well as removal of some bound moisture for NP, USP, MWP and IP, respectively (Table S2). A sharp peak at these temperatures demonstrated a sign of solid‐state changes, phase transitions, or crystalline rearrangement in relatively pure compounds (Kuznietsova et al. 2020). There was a broad endothermic peak at 111.92°C in FP (Table S2). The freezing procedure can change the physicochemical composition of tissues and disturb their structure (Ciurzyńska et al. 2021). This result may show the development of a supramolecular structure between the compounds in FP. Therefore, the thermal properties of FP were impacted. According to Δ*H* values, the pretreatments had a major impact on Δ*H*. While Δ*H* reduction was obtained by IP, Δ*H* was increased with FP. The reasons may be related that the melting enthalpy decreased as solids content increased. In addition, higher water content in solids may lead to enhance hydrogen bonding and requiring more energy to melt. Thus, melting enthalpy increased (Suresh et al. 2017).

## 4 Conclusion

The present study demonstrated that pretreatments significantly influenced the drying kinetics, physicochemical characteristics, and quality attributes of vacuum‐dried red beetroot slices. Among the investigated pretreatments, MWP provided the highest drying efficiency by accelerating moisture removal and reducing drying time. USP was more effective in preserving betalains and phenolic compounds. Pretreatments also improved color characteristics, rehydration ratio, reduced water activity and hardness, and promoted the formation of porous microstructures without substantially altering the functional groups of the samples. Furthermore, betalain compounds were successfully identified and quantified, confirming the potential of pretreatment‐assisted vacuum drying for producing high‐quality dried beetroot products. Overall, microwave and ultrasound pretreatments exhibited the most promising performance for producing high‐quality beetroot snacks. Although the developed products were intended as snack formulations, their sensory attributes and consumer acceptance were not evaluated. Therefore, future studies should include comprehensive sensory and consumer acceptance assessments and employ advanced chromatographic techniques to further elucidate compositional changes occurring during drying.

## Author Contributions

**Nuray İnan‐Çınkır:** project administration, supervision, writing – review and editing, writing – original draft, conceptualization, methodology, formal analysis, data curation, investigation, validation.

## Funding

This study was supported by Osmaniye Korkut Ata University (OKÜBAP‐2024‐PT1‐003).

## Conflicts of Interest

The author declares no conflicts of interest.

## Supporting information


**Table S1:** Thin layer drying models utilized for drying data.
**Table S2:** *T*_*o*_, *T*_*p*_, *T*_*e*_ and enthalpy (Δ*H*) values.
**Figure S1:** A representative chromatogram of betalain compounds (1: vulgaxanthin I, 2: betanin, 3: isobetanin).

## Data Availability

Data will be made available on request.
