# Supplementary material for: Effects of Different Pretreatments With Vacuum Drying on Drying Kinetics, Bioactive Properties and Physicochemical Characterization of Red Beetroot Snacks
Source: Food Sci Nutr. 2026 Aug 13;14(8):e72234. doi: 10.1002/fsn3.72234 (PMC13470033; doi:10.1002/fsn3.72234)
Supplement: Supplementary file 1 [file FSN3-14-e72234-s001.docx]

**Supplementary File**

Table S1. Thin layer drying models utilized for drying data

| **Model names** | **Equations** | **References** |
| --- | --- | --- |
| Page | MR = exp(-kt^n^) | Page (1949) |
| Modified page | MR = exp(-kt)^n^ | Overhults et al. (1973) |
| Approximation of diffusion | MR = a exp(-kt) + (1-a) exp(-kbt) | Toğrul and Pehlivan, (2004) |
| Sigmoid | MR = a + $\frac{b}{1+ e^{k(t-c)}}$ | Antal et al. (2011) |
| Logaritmic | MR = a exp(-kt) + c | Toğrul and Pehlivan, (2004) |
| Parabolic | MR = a + bt + ct^2^ | Sharma and Prasad (2004) |
| Cubic | MR = a + bt + ct^2^ + dt^3^ | Dalvand, Mohtasebi, and Rafiee (2012) |
| Vega Lemus | MR = (a + bt)^2^ | Vega Galvez et al. (2009) |
| Rational | MR = $\frac{a + bt}{1+ct+dt^{2}}$ | Haghi and Angiz (2007) |
| Vega Galvez 1 | MR = n + k$\sqrt{t}$ | Lemus-Mondaca et al. (2009) |
| Vega Galvez 2 | MR = exp(n + kt) | Lemus-Mondaca et al. (2009) |

MR: moisture ratio; a, b, c, d, k, k_o_, k_1_, n: model parameters; t: time.

Table S2. T_o_, T_p_, T_e_ and enthalpy (ΔH) values

|  | NP | | FP | IP | | MWP | | USP | |
| --- | --- | --- | --- | --- | --- | --- | --- | --- | --- |
|  | 1. peak | 2. peak | 1. peak | 1. peak | 2. peak | 1. peak | 2. peak | 1. peak | 2. peak |
| T_o_ (^o^C) | 29.29 | 159.33 | 58.13 | 57.16 | 172.24 | 56.13 | 159.54 | 28.28 | 150.88 |
| T_p_ (^o^C) | 75.35 | 175.67 | 111.92 | 98.99 | 182.5 | 94.68 | 174.32 | 76.04 | 170.38 |
| T_e_ (^o^C) | 107.34 | 183.57 | 188.08 | 128.89 | 190.25 | 129.99 | 182.28 | 101.13 | 185.45 |
| ΔH (J/g) | 51.57 | 38.66 | 214.34 | 46.90 | 5.72 | 56.13 | 40.88 | 57.14 | 59.95 |

T_o_: onset temperature, T_p_: peak temperature, T_e_: endset temperature

3

2

1

Figure S1. A representative chromatogram of betalain compounds (1: vulgaxanthin I , 2: betanin, 3: isobetanin)

**References**

1. Antal, T., Figiel, A., Kerekes, B., & Sikolya, L. (2011). Effect of drying methods on the quality of the essential oil of spearmint leaves (*Mentha spicata* L.). *Drying Technology, 29*(15), 1836–1844. <https://doi.org/10.1080/07373937.2011.606519>
2. Dalvand, M. J., Mohtasebi, S. S., & Rafiee, S. (2012). Determining the influence of drying conditions on the EHD drying process. *Journal of Agricultural and Biological Science, 7*, 396–401.
3. Haghi, A. K., & Angiz, F. Z. (2007). Heat and mass transfer in thermal drying of wool: A theoretical approach. In *Proceedings of the 5th Asia-Pacific Drying Conference* (pp. 443–448), Hong Kong.
4. Lemus-Mondaca, R., Miranda, M., Grau, A. A., Briones, V., Villalobos, R., & Vega-Gálvez, A. (2009). Effect of osmotic pretreatment on hot air drying kinetics and quality of Chilean papaya (*Carica pubescens*). *Drying Technology, 27*(10), 1105–1115. <https://doi.org/10.1080/07373930903221291>
5. Page, G. E. (1949). *Factors influencing the maximum rates of air drying shelled corn in thin layers* (Master's thesis, Department of Mechanical Engineering, Purdue University).
6. Overhults, D. G., White, G. M., Hamilton, H. E., & Ross, I. J. (1973). Drying soybeans with heated air. *Transactions of the ASAE, 16*(1), 112–113. <https://doi.org/10.13031/2013.37459>
7. Sharma, G. P., & Prasad, S. (2004). Effective moisture diffusivity of garlic cloves undergoing microwave-convective drying. *Journal of Food Engineering, 65*(4), 609–617. <https://doi.org/10.1016/j.jfoodeng.2004.02.027>
8. Toğrul, İ. T., & Pehlivan, D. (2004). Modelling of thin layer drying kinetics of some fruits under open-air sun drying process. *Journal of Food Engineering, 65*(3), 413–425. <https://doi.org/10.1016/j.jfoodeng.2004.02.001>
9. Vega-Gálvez, A., Notte-Cuello, E., Lemus-Mondaca, R., Zura, L., & Miranda, M. (2009). Mathematical modelling of mass transfer during the rehydration process of *Aloe vera* (*Aloe barbadensis* Miller). *Food and Bioproducts Processing, 87*(4), 254–260. <https://doi.org/10.1016/j.fbp.2008.10.004>
